# The utility of medico-legal databases for public health research: a systematic review of peer-reviewed publications using the National Coronial Information System

**DOI:** 10.1186/s12961-016-0096-1

**Published:** 2016-04-12

**Authors:** Lyndal Bugeja, Joseph E. Ibrahim, Noha Ferrah, Briony Murphy, Melissa Willoughby, David Ranson

**Affiliations:** Health Law & Ageing Research Unit, Department of Forensic Medicine, School of Public Health and Preventive Medicine, Monash University, 65 Kavanagh Street, Southbank, 3006 Australia; Coroners Court of Victoria, 65 Kavanagh Street, Southbank, Victoria 3006 Australia; Victorian Institute of Forensic Medicine, 65 Kavanagh Street, Southbank, 3006 Australia

**Keywords:** Coroners and medical examiners, Injury prevention, Mortality surveillance, National Coronial Information System Public health research

## Abstract

**Background:**

Medico-legal death investigations are a recognised data source for public health endeavours and its accessibility has increased following the development of electronic data systems. Despite time and cost savings, the strengths and limitations of this method and impact on research findings remain untested. This study examines this issue using the National Coronial Information System (NCIS).

**Methods:**

PubMed, ProQuest and Informit were searched to identify publications where the NCIS was used as a data source for research published during the period 2000–2014. A descriptive analysis was performed to describe the frequency and characteristics of the publications identified. A content analysis was performed to identify the nature and impact of strengths and limitations of the NCIS as reported by researchers.

**Results:**

Of the 106 publications included, 30 reported strengths and limitations, 37 reported limitations only, seven reported strengths only and 32 reported neither. The impact of the reported strengths of the NCIS was described in 14 publications, whilst 46 publications discussed the impacts of limitations. The NCIS was reported to be a reliable source of quality, detailed information with comprehensive coverage of deaths of interest, making it a powerful injury surveillance tool. Despite these strengths, researchers reported that open cases and missing information created the potential for selection and reporting biases and may preclude the identification and control of confounders.

**Conclusions:**

To ensure research results are valid and inform health policy, it is essential to consider and seek to overcome the limitations of data sources that may have an impact on results.

**Electronic supplementary material:**

The online version of this article (doi:10.1186/s12961-016-0096-1) contains supplementary material, which is available to authorized users.

## Background

Historically, the primary role of death investigation agencies was to inform the criminal justice system to establish whether the actions or inactions of another person had contributed to the death [1]. While there was early recognition that the outcomes of the death investigation process could also play a role in public health and safety [2], this has only been formally realised in a few jurisdictions across the world [3, 4]. To contribute to an evidence-base for public health and safety endeavours, electronic systems have been developed to store material and information generated for coroners and medical examiner’s medico-legal investigations of unexpected and unnatural deaths.

Such information comprises socio-demographic characteristics, medical history, circumstances immediately proximate to the death, the results of any forensic medical and scientific tests and the outcomes of legal proceedings. The richness of this population-based data makes medico-legal death investigation material a valuable data source for preventive medicine [5].

Increasingly, these data assume a wider use, enabling researchers to examine the nature, distribution and determinants of preventable deaths, and the effects of interventions [5–17]. This is of particular importance to policymakers and practitioners who have a responsibility for population-level health and safety [1, 12, 18]. These advances overcome some of the limitations of the time-consuming and costly traditional process of mortality data collection for public health research [10, 19]. It is more detailed and accurate than death certificates or police reports, and circumvents the need for extensive manual searches followed by review of all hard copy documents to identify cases and extract the information of interest [13, 20, 21].

Despite this, concerns remain about potential shortcomings of this dataset format, and the impact on research findings [22]. While electronic access reduces costs, and the time needed for case identification and data collection, discussion of the methodological limitations and strengths of this data for public health research remains rudimentary. The published research literature abounds with studies using different data sources and arriving at opposing conclusions [23]. Therefore, a failure to appreciate the presence and impact of limitations and strengths of data from medico-legal death investigations on research findings may lead to sub-optimal public health policy and intervention programs. In some cases, this may have significant clinical implications as illustrated by the shift in paradigm regarding hormone replacement therapy and risk of cardiovascular disease [24].

We explore this topic by examining the strengths and limitations reported in the published peer-reviewed literature by researchers who used medico-legal death investigation data in their study. We focussed on studies using the National Coronial Information System (NCIS), a national dataset for Australian and New Zealand coronial cases [25]. To date, evaluations of the NCIS focused on its utility as a tool for injury surveillance, and its completeness with ICD-10 coded data [26–29].

The aim of this narrative review was to identify and describe the strengths and limitations of a specific electronic data source for public health research as reported in peer reviewed original research publications, using the NCIS.

## Methods

### Definitions

The NCIS is an Internet-based data storage and retrieval system of all deaths reported to Coroners in Australia and New Zealand since 2000 and 2007, respectively [30]. It comprises coded and free-text data and up to four full text documents generated for the coroners’ investigation, namely the summary of text from the police report of death to the coroner, autopsy report, forensic toxicology report, and coroners’ findings.

### Search strategy

A systematic search, modelled on the Preferred Reporting Items for Systematic Reviews and Meta-Analyses (PRISMA) Statement [31], comprised a combination of electronic database searches, bibliographical reviews and literature referred by relevant experts. Electronic databases were searched for journal articles from the disciplines of medicine, law, public health, road safety and psychology for the period 1 July, 2000, to 31 July, 2014, restricted to the English language. The databases searched were PubMed, ProQuest and Informit using the search terms *NCIS* and *National Coroners Information System* (including the variations *Coroner’s*, *Coroners’* and *Coronial*). A general Internet search using the search engines Google and Google Scholar was also conducted to identify additional relevant publications. A list of publications was also provided by the NCIS.

### Article identification

The electronic database search strategy was intentionally broad to maximise the identification of relevant literature as the topic area crosses a number of disciplines. Results from search strategies were exported to EndNote for review and duplicates were removed. The reference lists of publications selected for the review were assessed against the inclusion criteria.

### Inclusion criteria

Publications were included in the review where the NCIS was a primary data source, was original research and was published in a peer-reviewed journal.

### Data collection

Methods to evaluate databases include self-reporting of limitations and strengths, or assessment by peers or external entities (e.g. Cochrane) based on a set of predetermined criteria [32]. We chose the former approach on the premise that strengths and limitations would be identified via the peer-review process [33]. Three authors (author 1, author 4 and author 5) independently reviewed the full text of each publication that met the inclusion criteria and extracted the following information in a Microsoft Excel spreadsheet: author(s); date published; title; death or injury type; publication type; publication name; peer review status; and reported strengths and limitations of the NCIS as a data source (free text). Any disagreement in the classification of a variable was resolved by discussion with a fourth researcher (author 2). If insufficient information was available, the variable was classified as unknown.

### Data analysis

A descriptive statistical analysis was conducted to describe frequencies of published original research, subject of research conducted, and studies that included a discussion of the strengths and limitations of the NCIS as a data source and the impact on their findings. Authors 1, 4 and 3 independently conducted a content analysis of this information to identify the nature, impact of strengths and limitations of the NCIS as reported by researchers.

## Results

### Study selection

**Fig. 1:**
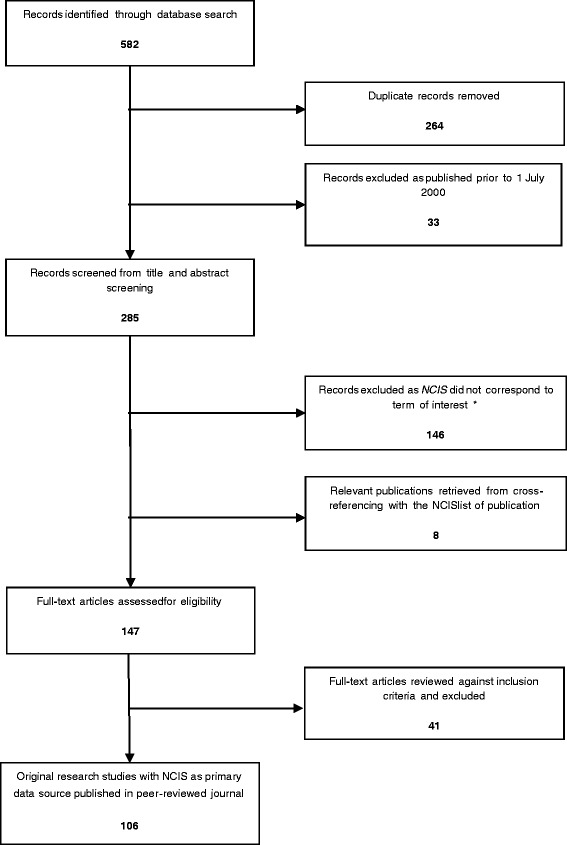
PRISMA Flow diagram of identification, screening, and inclusion of eligible articles. * The search term *NCIS* is also an acronym for other organisations and scientific terminology

The literature searches and cross-referencing with the NCIS list of publications yielded 147 studies, of which 106 were included (Fig. 1; see descriptive table of studies in Additional file 1).

### Study description

#### Journals

The majority of studies were published in Australia (41/106, 38.7%) and the United Kingdom (34/106, 32.1%), most commonly in the fields of occupational health and safety (28/106, 26.4%) and medicine (26/106, 24.5%) (Table 1). Most journals ranked in the first or second quartile (64/106, 60.3%) within their category, although approximately 20% (20/106) of journals were not indexed (Table 1).

**Table 1:** Description of journals and publications reviewed (*n* = 106)

	Number of publications	Proportion of publications
	*n*	%
Journal country		
Australia	41	38.7
United Kingdom	34	32.1
United States of America/Canada	14	13.2
Germany	6	5.7
Ireland	5	4.7
Switzerland	3	2.8
Netherlands	2	1.9
United Arab Emirates	1	0.9
Journal category		
Occupational Health and Safety	28	26.4
Medicine	26	24.5
Law and Legal Medicine	13	12.3
Psychiatry	12	11.3
Health policy & services	9	8.5
Pharmacology	7	6.6
Engineering	7	6.6
Other^a^	4	3.8
Journal ranking quartile within category		
Q1	33	31.1
Q2	31	29.2
Q3	12	11.3
Q4	10	9.4
Not indexed	20	19.0
Design		
Retrospective case series	94	88.7
Retrospective cohort	6	5.7
Ecological	5	4.7
Prospective case series	1	0.9
Number of years of data		
<2 years	5	4.7
2–5 years	18	17.0
6–9 years	62	58.5
≥10 years	19	17.9
Unknown	2	1.9
Intent		
Unintentional	42	39.7
All intent	35	33.0
Intentional self-harm	27	25.5
Assault	1	0.9
Natural	1	0.9
Death type		
Intentional self-harm	27	25.5
Transport	18	17.0
Other reportable deaths^b^	13	12.3
Toxicology	12	11.3
Work	10	9.4
Special groups^c^	8	7.5
Drowning	7	6.6
Recreation	7	6.6
Location^d^	4	3.8

^a^Includes multidisciplinary sciences, sport sciences and social sciences

^b^Includes natural deaths, assaults, traumatic brain injuries and other unintentional deaths

^c^Includes children, Indigenous and Torres Straight Islanders, and prisoners

^d^Includes farms, emergency departments and residential aged care

#### Study design

As expected, in nearly all studies (94/106, 88.7%), the research design comprised a retrospective case series of deaths (Table 1). Almost half (50/106, 47.2%) of studies examined data at a national level, and 46 (43.4%) in a single jurisdiction. The annual frequency of publications steadily increased from one in 2003 [34] to 18 in 2013. Where known (n = 104), the number of years of NCIS data examined ranged from 1 to 13 years (median = 7 years; IQR, 6–9; Table 1).

#### Types of death

The majority of studies examined unintentional deaths (42/106, 39.7%) and, interestingly, only two investigated assaults and natural deaths (Table 1). Within specific death types, studies most commonly investigated intentional self-harm (27/106, 25.5%) and deaths resulting from a transport collision (18/106, 17.0%; Table 1).

### Reporting of the strengths and limitations of the NCIS

Thirty (28.3%) studies reported strengths and limitations, 37 (34.9%) limitations only and seven (6.6%) strengths only. Fourteen (13.2%) studies discussed the impacts of the strengths of the NCIS, whilst 46 (43.4%) discussed the impacts of limitations.

#### Strengths

The reported strengths of the NCIS were classified in five domains (Table 2).

**Table 2:** Strength domains and reported impacts

Domain – Information was …	Number of publications (proportions)^a^	Justifications	Reported impacts
COMPREHENSIVE			
Comprehensive coverage	16 (43%)	Captures all reportable deaths across Australia and New Zealand Wide breadth of information in closed cases	Monitor mortality trends at population-level Inform injury prevention programs and priorities Assess effectiveness of interventions
DETAILED			
Detailed data source	13 (35%)	Richness of information in closed cases Greater level of detail than existing databases (e.g. Australian Bureau of Statistics)	Detailed examination of causes and circumstances of death
RELIABLE			
Data consistency	11 (29%)	Quality assessment by trained staff at the National Coronial Information System (NCIS) and internal quality control Consistent with other datasets and national statistics More reliable than national statistics wherein data are finalised before coroner’s investigation is closed	Accurate estimate of mortality
Rigorous coding framework		Rigorous and consistent coding scheme	
APPLICABLE			
Utility for death investigation Utility for public health and safety and injury prevention	9 (24%)	Hazard identification tool Coroner’s inquest yields constructive recommendations	Potential for reduction in preventable deaths Valuable surveillance tool
OF HIGH QUALITY			
Good quality data	6 (16%)	Valid information Quality assessment by trained staff at NCIS and internal quality control	Contribute to validity of study findings
CURRENT			
Most current data source available	4 (10%)	Contemporary information Timely access	Not specified

^a^Proportions add up to over 100% as studies may report multiple categories of strengths

##### Comprehensive coverage (*n* = 16; 43%)

The most frequently reported strength of NCIS was comprehensive coverage. Studies reported that all or the majority of relevant cases were stored in the NCIS, across variables including indigenous origin, objects or substances producing injury and mechanism of injury. The NCIS was described as the only data source that stores information on all work-related deaths irrespective of employment status and setting [35].

##### High level of detail (*n* = 13; 35%)

The NCIS was reported to hold more detailed information compared with other mortality datasets such as the Australian Bureau of Statistics [36], particularly regarding causes and circumstances of death [37]. Studies reported that this was in part due to the accessibility of full text documents [38].

##### Reliability (*n* = 11; 29%)

Reliability was described as a strength of the NCIS and demonstrated by its consistency with other data sources, such as workers compensation claims [39] and official national statistics [36]. Additionally, studies noted that rigorous coding systems and internal quality assessment operated by the NCIS [40] ensured that the information stored reliably matched that of deaths reportable to coroners [41].

##### Utility (*n* = 9; 24%)

The NCIS was described as a useful tool for death investigation and research on public health and safety. Notably, access to coroners’ recommendations was reported to be particularly useful [42].

##### High data quality (*n* = 6; 16%)

The NCIS was reported as containing high quality data, albeit with little substantiation provided.

##### Timeliness (*n* = 4; 10%)

Timeliness of data was the least often mentioned strength [43].

#### Impacts of strengths

Reported impacts included the ability to identify and monitor trends in specific death types [27, 39, 44, 45]. Moreover, NCIS may be of valuable use to both researchers [46] and injury prevention practitioners/policymakers [38], to identify hazards [29, 47], inform the development of prevention strategies [27, 36, 39, 48], and assess their effectiveness [49]. In addition, detailed data enabled the in-depth investigation of specific mechanisms of death, such as drowning [38, 50], or activities such as work relatedness [51]. Finally, the reliability and quality of the data enabled the accurate estimation of mortality [48, 52].

#### Limitations

Reported limitations of the NCIS were classified in three main domains (Table 3).

**Table 3:** Limitation domains, remediation and reported impacts

Domain – Information was not …	Number of publications (proportions)^a^	Reported impact on findings	Actions taken
AVAILABLE			
Open cases	23 (34%)	Under-reporting of potentially relevant cases	Exclusion of all open cases Exclusion of recent deaths
		Inability to verify information	Used additional data source
Whole documents/information of interest	26 (39%)	Unable to conduct detailed analysis	Access paper-based record Adjust analysis Cases not categorised
		Under-reporting of potentially relevant cases	Exclusion of cases
Small dataset	2 (3%)	Unable to detect trends and evaluate impacts of interventions	Acknowledged limitation
COMPLETE			
Missing data on available info of interest	16 (24%)	Missing/incomplete data for analysis Limitation of level of analysis	Access paper-based record Cases not categorised Variables excluded from analyses Used additional data source Acknowledged uncertainty (absence of data does not mean factor not associated)
ACCURATE			
Potential for human error	5 (7%)	Errors in information recording during inquest (e.g. spelling of drug names)	Not specified
Potential for human error in coding	7 (10%)	Missing data for some variables Misclassification of variables Affects accuracy of NCIS	Access paper-based record Used additional data source Careful interpretation of reports
Misclassification of intent re: intentional self-harm	5 (7%)	Under-reporting of potentially relevant cases	Used additional data source
Discrepancies between the National Coronial Information System (NCIS) and ICD-10	3 (4%)	Under-reporting of potentially relevant cases	Used additional data source (ICD-10)

^a^Proportions add up to over 100% as studies may report multiple categories of limitations

##### Lack of availability (*n* = 51; 76%)

Unavailability of data of interest was the most frequently reported limitation, most commonly within deaths still under investigation (open cases). For records that were available, studies reported that some or all of the full text documents were not attached, and that this varied between jurisdictions [38, 53]. Lack of availability was also mentioned in relation to data items not routinely recorded in the NCIS and/or by the coroner, such as past history of imprisonment [37], clinical histories [54, 55], and whether inquests were mandatory or discretionary [56].

##### Completeness (*n* = 16; 24%)

Studies reported that details on historical and proximate circumstances of death were missing from available documents [50, 57, 58]. This precluded a detailed analysis to identify contributing factors to injury (Table 3). Completeness again differed between jurisdictions [38], and while some reports provided detailed descriptions, others contained only minimal information. It was therefore not possible to ascertain from the data stored in the NCIS whether the absence of information meant that it was truly not present or simply omitted [59]. Finally, it was noted that missing information was most prominent between 2000 and 2006 [60].

##### Accuracy (*n* = 20; 28%)

Studies reported that information in the NCIS may be inaccurate due to coding errors [61–63]. Whilst it was noted that the NCIS conducts a data quality program to review and correct information, this cannot be applied to all records [64]. Additionally, discrepancies between the NCIS and ICD-10 classification system reportedly resulted in a different number of retrieved cases depending on the code-set used [27, 29].

#### Impacts of limitations

Impacts of limitations consisted primarily of under-reporting of relevant cases, incomplete datasets, misclassification, and the inability to detect trends (Table 3). Studies reported implementing a number of measures to mitigate these shortcomings, including exclusion of open cases or those with missing information, seeking access to paper-based records or other source of information, or adjusting the analysis (Table 3). The exclusion of open cases was achieved in two ways: for all open cases within each year of the study, or for all cases in the last two years of a given study period owing to the delay in case closure [48, 65–68]. Studies acknowledged that this may result in erroneously decreasing trends in mortality where the overall number of cases is small [49, 69], and in selection bias [9] or reporting bias [70] as investigations of deaths that occurred in unequivocal circumstances may be more rapidly completed. As a result, a review of preliminary information was conducted in several studies to identify variables that may be associated with a systematic bias [71]. In addition, analyses both including and excluding open cases reached equivalent results [36], suggesting that excluding open cases does not introduce bias [65]. Furthermore, it was suggested that the effect, if any, was likely to be small due to the overall small number of cases excluded [9]. However, estimation of under-reported cases ranged from 8% to 25% [49, 57, 72], which may also reduce the sample size and statistical power.

Consequences of missing information on the deceased and circumstances surrounding death rather than on direct causes of death were two-fold. Firstly, case identification was undermined, leading to under-reporting and the inability to monitor mortality trends [37]. A study reported that the NCIS only identified a small subset of reportable deaths amongst ex-prisoners [45]. Secondly, detailed analysis of mechanisms of injury and identification of contributing factors were not always possible. Accordingly, access to paper records was sought [57] and text findings reviewed [56]. Another measure entailed adjusting analysis. In one study, all cases of child fatalities with unspecified family structure were assigned as from an intact biological family [70]; others excluded incomplete cases [60]. Finally, some studies reported the dataset to be too small to monitor trends as it stores cases from 2000, when the NCIS started operating [47, 73].

## Discussion

### Summary of results

To illustrate the benefits and hazards of using medico-legal databases in health policy research, this study described the strengths and limitations of the NCIS, as identified in published peer-reviewed studies that used this dataset. The NCIS was reported to be a reliable source of high quality, detailed information with comprehensive coverage of deaths of interest, making it a powerful surveillance and hazard identification tool that enables the examination of spatiotemporal trends, while providing sufficient detail to examine specific processes and outcomes in-depth. The NCIS also averts the previously laborious, slow and costly process of accessing mortality data.

Despite these strengths, studies reported that open cases and missing information created the potential for selection and reporting biases and may preclude the identification of confounders [32]. Our findings are consistent with previous studies examining coronial [74, 75] and other health databases [76, 77].

### Strengths and limitations

To our knowledge, this is the first study to ascertain the strengths and limitations of the NCIS as determined by the researchers and their scientific peers. Overall, studies were published in a broad range of journals in terms of country of origin, field and level of expertise, which contributes to the generalizability of the results. In addition, a broad and systematic research strategy was employed to identify eligible studies, and a search within the NCIS’ own database ensured published studies were not missed. Studies published in peer-reviewed journals were included on the premise that their findings had been subject to the imperfect yet indispensable peer-review process, thus conferring them legitimacy [78, 79].

This study has a number of limitations. Restriction to peer-reviewed publications led to the exclusion of a number of studies utilising NCIS and published in the grey literature. Additionally, peer-reviewed journals have a number of shortcomings. The exponential increase in manuscripts submitted for publication, coupled with the lack of qualified referees, may result in the publication of studies of substandard quality [80]. Peer-reviewed journals are also susceptible to selective publication bias, whereby studies that fail to demonstrate a significant difference or do not confirm previous results are not published [81]. Reviewers may preclude the appearance and dissemination of findings if these do not fit their own beliefs [82].

Finally, only self-reported biases in selected studies were discussed. Other biases may be associated with the NCIS, yet were not reported. In addition, and consistent with previous studies [83], the majority of studies did not discuss the impacts of limitations on their findings. Authors may have a vested interest in minimising limitations in order to optimise their chance of publication [23].

### Implications

Prior to the emergence of medico-legal databases, the study of unexpected and unnatural deaths primarily consisted of traditional surveillance programs led by government agencies with a public health or safety focus that usually examined specific death types such as deaths in custody. However, their conclusions were limited by incomplete data obtained based on voluntary reporting from local agencies, or by crude information from national statistics such as the National Vital Statistics System in the United States or the Australian Bureau of Statistics in Australia [84].

Population-based health information systems, such as cancer registries, have been used for decades [85] and are becoming increasingly common [76, 77]. To ensure the outcomes of studies that draw on information from medico-legal databases are valuable and reliably translated, policymakers ought to be aware of their strengths and limitations such as the potential underestimation of suicides. Importantly, datasets such as the NCIS were not designed with the sole purpose of academic research or with specific research questions in mind [76]. As a result, variability in the content and detail of information they contain is a function of the variability within and between coroners and coronial jurisdictions.

A number of recommendations to manage the pitfalls of medico-legal databases emerged from our findings. Researchers should estimate the number of open cases, consider how their exclusion may introduce bias, and take measures to overcome the impact of bias. Additional data sources may optimise accurate case identification and validate information of interest. Consideration should also be given to linkage with other datasets, such as healthcare or employment records [86].

Finally, our findings highlight that overall users of datasets include scant information regarding the biases inherent to their data source. Therefore, another approach to evaluating the quality of medico-legal datasets may be using a systematic criteria-based or program evaluation. This includes assessing whether the whole dataset or certain elements are consistent with another source, whether expected data elements are present, and whether summary statistics derived from the database fit the expected distribution for a given clinical concept [87].

### Future directions

Our findings can be generalised to other medico-legal databases in countries with comparable public health systems, be applied by researchers to determine whether a given database is a suitable data source to address a particular question, and may inform the design of their study.

The NCIS is the first national database of medico-legal information worldwide. Similar databases exist in the United States (National Violent Death Reporting System) and Canada (Canadian Coroners and Medical Examiner Database). However, they do not capture all unexpected and unnatural deaths nationwide. National databases are cost-effective [88], reduce the bureaucracy associated with conducting research and surveillance initiatives, and may avoid setbacks such as the failure to establish a surveillance program for Creutzfeldt-Jacob disease based on medico-legal data in the United Kingdom due to concerns regarding the independence of Coroners [6]. The development of national medico-legal databases is bound to grow, and our findings will inform their design. The quality of any dataset could be improved; however, this usually involves investment of resources creating a tension between the cost of data and the quality.

## Conclusions

The choice of data source may significantly impact results of studies, and leads to sometimes contradictory conclusions. Using the case of the NCIS, this study identifies the key strengths and limitations of a national medico-legal database as used by public health researchers and vetted by their peers. This information allows researchers, policymakers and practitioners to be better prepared when considering whether a research question is addressed by a given data source and the validity of the study outcome. Hopefully, this will reduce waste of precious resources and result in better public health initiatives.

## Additional file

Additional file 1: Descriptive table of original research studies with NCIS as primary data source published in peer-reviewed journal. (PDF 274 kb)
