# Supplementary material for: The utility of medico-legal databases for public health research: a systematic review of peer-reviewed publications using the National Coronial Information System
Source: Health Res Policy Syst. 2016 Apr 12;14:28. doi: 10.1186/s12961-016-0096-1 (PMC4828834; doi:10.1186/s12961-016-0096-1)
Supplement: Additional file 1: — Descriptive table of original research studies with NCIS as primary data source published in peer-reviewed journal. (PDF 274 kb) [file 12961_2016_96_MOESM1_ESM.pdf]

Additional files

Additional file 1: Descriptive table of original research studies with NCIS as primary data source published in peer-reviewed journal

Author, Year	Journal	Country of Journal	Death Type	Aim	Study Design	Study Period	Jurisdiction	Population	NCIS as Data Source	Main Findings
Woolfenden, 2003	Journal of Paediatric and Child Health	Aus	All deaths	To examine characteristics and death of children presenting to emergency departments.	Retrospective chart review	2	NSW	Children	2	Presentation to emergency departments with aggression is an indicator of significant psychosocial dysfunction.
Driscoll, 2004	International Journal of Injury Control and Safety Promotion	UK	Drowning	To examine the contribution of alcohol to drowning deaths.	Retrospective case series	2	Multiple	All	Dual 1	High blood alcohol levels are less common in drowning deaths than previously but still frequent.
Freckelton, 2005	Psychiatry, Psychology and Law	UK	Intentional self-harm	To review coroner's recommendations on suicides amongst individuals with psychiatric disorders.	Retrospective case series	6	National	All	1	Coroners' recommendations should be more accessible and mandatory.
Tooley, 2006	Evolution and Human Behaviour	US	Injury-related death	To investigate the association between living with a step parent and unintentional childhood fatal injury.	Retrospective case series	4	Multiple	Children	1	Step children are at significantly increased risk of unintentional fatal injury
Daking, 2007	Health Information Management Journal	Aus	All reportable	To compare data coded to ICD-10 between ABS and NCIS.	Retrospective case series	4	National	All	Dual 1	Discrepancies between the two sets of codes were detected in over 50% of cases.
Hanssens, 2007	Aboriginal and Islander Health Worker Journal	Aus	Intentional self-harm	To identify contributing factors for clustering of suicide in NT.	Retrospective case series	5	NT	Indigenous	Dual 1	Evidence of "clustering" and "echo clusters" (communities do not recover before next cluster of suicide).
Hanssens, 2007	Aboriginal and Islander Health Worker Journal	Aus	Intentional self-harm	To investigate the contribution of a contagion effect, alcohol and familiarity with hanging as risk factors for Indigenous suicide.	Retrospective case series	7	NT	Indigenous	Dual 1	Evidence of contagion , alcohol and hanging risk factors operating within clusters of suicide
MacBean, 2007	Medical Journal of Australia	Aus	Bite-related deaths	To describe the epidemiology of human and non-human bite	Retrospective case series	7	VIC	All	2	Mammal bite injuries are common and often require

Author, Year	Journal	Country of Journal	Death Type	Aim	Study Design	Study Period	Jurisdiction	Population	NCIS as Data Source	Main Findings
				injuries.						inpatient care.
Driscoll, 2008	Journal of Safety Research	US	Work-related deaths	To assess the contribution of design to work-related deaths in Australia.	Retrospective case series	3	National	All	1	Design is a significant contributor to work-related fatal injury.
Fragar, 2008.	Journal of Occupational Health and Safety - Australia and New Zealand	Aus	Unintentional deaths	To examine trends on non-intentional injury deaths in farms.	Retrospective case series	4	National	Farmers	1	Reduction in number and change in profile of farm unintentional deaths.
Hanssens, 2008	Aboriginal and Islander Health Worker Journal	Aus	Intentional deaths	To investigate the contribution of imitation and contagion in rise of Indigenous suicide rate.	Retrospective case series	8	NT	Indigenous	Dual 1	Significant space-time-method clustering provides indirect evidence of imitative suicide and contagion.
Kaye, 2008	Addiction	UK	Drug-related deaths	To investigate the pathology of methamphetamine related deaths.	Retrospective case series	6	National	All	1	Methamphetamine has contributed to a substantial number of deaths in Australia, particularly via its cardiotoxicity and use with other drugs.
Watterson, 2008	Australian Indigenous Law Review	Aus	Reportable deaths	To examine coronial recommendations in Indigenous deaths and their implementation.	Retrospective case series	3	National	Indigenous	1	There were significant inconsistencies in the treatment of death in custody. Coronial recommendation can impact incidence of death but have been overlooked.
Young, 2008	Conference proceedings	Aus	Transport-related deaths	To investigate the types of information required to examine crashes.	Retrospective case series	7	National	All	1	Low coroners' reports and key case indicators were the most important limitations.
Bell, 2009	Drug and Alcohol Dependence	Switzerland	Poisoning	To investigate overdose mortality (in-treatment and diversion) with methadone and buprenorphine.	Retrospective case series	1	NSW	All	1	Buprenorphine is associated with lower overdose risk than methadone.
Brodie, 2009[1]	Accident Analysis and Prevention	UK	Transport-related unintentional deaths	To describe the nature and extent of heavy vehicle crashes.	Retrospective case series	6	VIC	All	Dual 1	Crashes involved a single vehicle leaving the roadway on a straight road, stimulants or cannabis, excess speed and absence of

Author, Year	Journal	Country of Journal	Death Type	Aim	Study Design	Study Period	Jurisdiction	Population	NCIS as Data Source	Main Findings
										seatbelt
Elnour, 2009	Australian and New Zealand Journal of Public Health	Aus	Intentional deaths	To compare intentional deaths in ABS and NCIS datasets.	Retrospective case series	6	National	All	1	Underestimation of suicide mortality in Australia due to incomplete coroner data available to ABS coders.
Grzebieta, 2009	Journal of the Australasian College of Road Safety	Aus	Transport-related deaths	To examine motorcycle impacts into any roadside barrier.	Retrospective case series	7	National	All	1	The number of motorcycle impacts into roadside barrier was low, and predominantly involved W-beam steel barriers.
Hiller, 2010	Emergency Medicine Journal	UK	Unintentional deaths	To investigate the nature, management and outcomes of traumatic aortic transection.	Retrospective case series	2	VIC	All	Dual 1	Aortic transection was most commonly due to road crashes affecting young men, with a mortality rate of over 90%.
Kaye, 2009	Drug and Alcohol Dependence	Switzerland	Drug-related deaths	To characterise MDMA-related deaths in Australia.	Retrospective case series	6	National	All	1	Methamphetamine has contributed to a substantial number of deaths in Australia, particularly via its cardiotoxicity and use with other drugs.
Morgan, 2009	Journal of Science and Medicine in Sport	Aus	Drowning deaths	To develop a direct observation measure of frequency and duration for surf beach bather exposure to water.	Prospective case series	2 months	VIC	All	Dual 1	Overrepresentation of adult males in surf beach drowning statistics is partly due to greater exposure to deeper water
Pridmore, 2009	Australian and New Zealand Journal of Psychiatry	UK	Intentional deaths	To characterize suicide in the NT.	Retrospective case series	7	National	Indigenous	Dual 1	Suicide is more common in NT generally, and more common in NT Indigenous than NT non-Indigenous people.
Samarasekera, 2009	Journal of Paediatric and Child Health	Aus	Chest trauma deaths	To characterise the nature and management of major paediatric chest trauma	Population-based study	7	VIC	Children	2	Serious injury requiring surgical intervention is rare.
Sikic, 2009	Medical Journal of Australia	Aus	Transport-related deaths	To investigate the incidence and nature of bicycling injuries	Retrospective case series	6	VIC	All	Dual 1	Incidence has significantly increased in the last few years.
Sturgiss, 2009	Journal of Forensic and Legal Medicine	UK	Intentional deaths	To characterise suicide in people over the age of 65 years.	Retrospective case series	7	ACT	People aged > 65 years old	1	Suicide by hanging is not an uncommon mechanism in people over the age of 65 years.

Author, Year	Journal	Country of Journal	Death Type	Aim	Study Design	Study Period	Jurisdiction	Population	NCIS as Data Source	Main Findings
Barron, 2010	Police Practice and Research	UK	Intentional deaths	To characterise the personal, occupational, psychological, and social characteristics of police officers who commit suicide.	Retrospective case series	9	NSW	Police force	2	Risk factors are similar to general community.
Brodie, 2010[1]	Australian and New Zealand Journal of Public Health	Aus	Transport-related deaths	To describe the nature of coroners' recommendations on fatal heavy vehicle crashes	Retrospective case series	7	VIC	All	1	Coroners' recommendations varied by vehicle type and number of fatalities.
Brodie, 2010[1]	Injury Prevention	UK	Work and recreational-related deaths	To examine fatalities from tree felling.	Retrospective case series	8	VIC	All	1	70% of fatal accidents involved persons not employed within a relevant industry.
Bugeja, 2010[1]	Health Information Management Journal	Aus	External causes of death	To quantify the completeness of external cause ICD-10 codes in the NCIS compared with ABS.	Retrospective case series	6	VIC	All	1	4,397 (70.8%) matched exactly with the independently assigned ICD-10 code. Disparity primarily related to intent and specificity.
Cutajar, 2010	Medical Journal of Australia	Aus	Poisoning deaths	To determine the rate and risk of intentional and unintentional drug overdose in individuals sexually abused in childhood.	Cohort study	9	VIC	All	Dual 1	Child sexual abuse victims are at increased risk of suicide and accidental fatal drug overdose.
Franklin, 2010.	Medical Journal of Australia	Aus	Drowning deaths	To examine trends in drowning deaths characteristics, and the feasibility of achieving a 50% reduction in unintentional drowning deaths by 2020.	Retrospective case series	6	National	All	1	A 50% reduction in drowning fatalities may be achievable in certain types of immersions.
McNeilly, 2010[1]	Injury Prevention	UK	Work-related death	To determine the presence of alcohol, cannabis and amphetamine-type stimulants in work-related injury deaths.	Retrospective case series	6	VIC	All	1	Alcohol and/or drugs were present in a significant portion of work-related deaths, which were mainly transport-related.
Mikocka-Walus, 2010	Emergency Medicine Australasia	Aus	Transport-related death	To compare the on- and off-road motorcycle-related major trauma.	Retrospective case series	8	VIC	All	Dual 1	Off-road motorcycle-related major trauma has increased, whilst on-road motorcycle-related death has decreased.

Author, Year	Journal	Country of Journal	Death Type	Aim	Study Design	Study Period	Jurisdiction	Population	NCIS as Data Source	Main Findings
Mitra, 2010	Emergency Medicine Australasia	Aus	Transport-related death	To determine the incidence and pattern of injuries in tram-related injuries.	Retrospective case series	8	VIC	All	Dual 1	Although most cases involved minor injuries, there was a significant increase in the incidence of major trauma cases, mostly pedestrians.
Pilgrim, 2010	Forensic Science International	Ireland	Poisoning deaths	To determine the involvement of selected serotonin-active drugs in sudden death.	Retrospective case series	7	VIC	All	1	
Rintoul, 2010	Injury Prevention	UK	Poisoning deaths	To describe the trends and demographic characteristics in fatal drug toxicity involving oxycodone.	Population-based observational study	10	VIC	All	1	
Robinson, 2010	Psychiatric Research	UK	All reportable	To determine the rate of sudden death and socio-demographic characteristics among young people with first episode psychosis following commencement of treatment.	Cohort study	9	National	Young people	Dual 1	Of nineteen deaths, most were suicide. Risk factors were male gender, previous attempt and greater symptom severity.
Studdert, 2010	Medical Journal of Australia	Aus	All reportable	To evaluate the changes in the understanding of the manner and cause of death occurring during the course of coronial investigations.	Ecological analysis	9	National	All	1	Coronial investigations transform basic understanding of cause of death in only a small minority of cases.
Andrews, 2011	BMC Research Notes	UK	All reportable	To determine the proportion of reportable deaths among ex-prisoners.	Retrospective case series	7	National	Ex-prisoners	1	Only a small minority of reportable deaths among ex-prisoners was detected.
Austin, 2011	Journal of Forensic Sciences	US	Intentional self-harm	To investigate the cases of suicide involving helium inhalation.	Retrospective case series	9	National	All	1	There was a recent and striking increase in cases of suicide involving helium inhalation.
Beacroft, 2011	Australian Indigenous Law Review	Aus	Deaths in custody	To examine trends and issues in deaths in custody.	Retrospective case series	9	National	Indigenous	Dual 1	A greater proportion of deaths in police custody involves Indigenous than non-Indigenous Australians.

Author, Year	Journal	Country of Journal	Death Type	Aim	Study Design	Study Period	Jurisdiction	Population	NCIS as Data Source	Main Findings
Bugeja, 2012[1]	Injury Prevention	UK	All reportable	To quantify and examine coroners' recommendations according to principles of injury prevention.	Cohort study	6	VIC	All	1	Coroners' recommendations were relatively rare, and did not systematically draw on injury prevention research.
Dearn, 2011	Journal of Law and Medicine	Aus	All reportable	To examine the factors contributing to the "preventable deaths" of people under 50 years of age in residential aged care.	Case study	6	VIC	Aged Care residents	Dual 1	Of the three cases of "preventable" deaths, none was reported, and all have implications for systemic reform.
Franklin, 2011	Journal of Paediatric and Child Health	Aus	Drowning deaths	To characterise the immersion rescuer–victim dyad where the primary 'victim' is a child and the 'rescuer' drowns.	Retrospective case series	6	National	Children	1	The dyad involves a male, parent, partner or relative; an unfamiliar water hazard; a tourist; the primary victim usually survives.
Frechede, 2011	Accident Analysis and Prevention	UK	Transport-related deaths	To compare the relative contribution, cause of death and risk factors of all single vehicle crash types between 3 States.	Retrospective case series	8	Multiple	All	1	Rollovers accounted for a third of all occupant fatalities, with differences in mechanisms, crash type and containment of the occupant between states.
Jama, 2011	Accident Analysis and Prevention	UK	Transport-related deaths	To describe and compare motorcycle-into-barrier crashes in Australia and New Zealand.	Retrospective case series	6	National	All	1	Fatalities usually involved a single vehicles, young men, steel W-beams, speeding and high blood alcohol levels. A majority occurred on a weekend, during daylight hours, on clear days with dry road surfaces.
Jones, 2011[1]	Injury Prevention	UK	Work-related deaths	To describe the nature and mechanisms of work-related non-crash heavy vehicle driver fatalities.	Retrospective case series	10	National	All	Dual 1	Forty-seven cases of a truck driver being killed while performing a non-driving task were identified from the NCIS. A common sequence of events was identified in 21 (45%) cases involved parking on an incline combined with brakes not functioning appropriately leading to a crushing death.

Author, Year	Journal	Country of Journal	Death Type	Aim	Study Design	Study Period	Jurisdiction	Population	NCIS as Data Source	Main Findings
Petrass, 2011	Medical Journal of Australia.	Aus	Drowning deaths	To determine the frequency of lack of supervision in child drowning.	Retrospective case series	10	National	Children	1	Lack of supervision was identified as a contributing factor in most cases.
Pilgrim, 2011	International Journal of Legal Medicine	Germany	Poisoning deaths	To examine the incidence of inappropriate prescribing patterns involving serotonergic drugs.	Retrospective case series	7	VIC	All	1	Cases comprised contraindicated drug combinations, mostly a combination of three to five drugs.
Pilgrim, 2011	Journal of Analytical Toxicology	US	Poisoning deaths	To determine the incidence of ecstasy use in drug interaction related sudden death.	Retrospective case series	7	VIC	All	1	Half of cases involved the concomitant use of MDMA with other drugs that were likely to result in an adverse drug reaction.
Roxburgh, 2011	Medical Journal of Australia	Aus	Poisoning deaths	To document trends in morphine and oxycodone prescription, hospital separations for overdose, adverse events and mortality.	Cross-sectional study	9	National	All	Dual 1	Oxycodone prescriptions have increased, particularly among older Australians.
Andrew, 2012	Injury	UK	Recreational deaths	To examine trends in sport and recreation activities-related deaths.	Retrospective case series	7	VIC	All	1	There was an increase in the rate of major trauma or death from sport and active recreation injuries.
Andrews, 2012	BMC Public Health	UK	Poisoning deaths	To explore the causes and circumstances of drug-related mortality among ex-prisoners.	Retrospective case series	8	National		1	Drug-related deaths are common among ex-prisoners and often occur in a home, with use of multiple substances.
Bambach, 2012	Accident Analysis and Prevention	UK	Transport-related deaths	To analyse injury profiles in motorcycle-barrier crashes.	Retrospective case series	6	National	All	1	The thorax region had the highest incidence of injury followed by the head.
Byard, 2012	Journal of Forensic Sciences	US	Recreational deaths	To characterise autoerotic death or sexual asphyxia.	Retrospective case series	7	National	All	Dual 1	Lethal sexual asphyxia is uncommon in Australia and Sweden, with a young male predominance.
Cheung, 2012	Social Science & Medicine	UK	Intentional self-harm	To identify sex-specific suicide pattern and metropolitan-rural-remote differentials.	Retrospective case series	5	National	All	1	There were rates of suicide in rural and remote areas for males, with some high risk postal areas. Socio-economic deprivation,

Author, Year	Journal	Country of Journal	Death Type	Aim	Study Design	Study Period	Jurisdiction	Population	NCIS as Data Source	Main Findings
										Indigenous status and low access to mental health service were risk factors.
Curran, 2012	Emergency Medicine Australasia	Aus	Deaths in emergency departments	To determine recurrent themes among coroners' recommendations aiming at increase the safety of ED care.	Retrospective case series	2	Multiple	All	1	Recommendation themes related to issues of risk management/medico-legal, diagnostic/therapeutic error, education, documentation/communication and re-presentation.
Deasy, 2012	Resuscitation	Ireland	Injury-related deaths	To describe the profile of paediatric traumatic out of hospital cardiac arrest.	Retrospective case series	10	VIC	Children	Dual 1	Paediatric traumatic out of hospital cardiac arrest is uncommon. Resuscitation efforts are seldom effective and associated with poor neurological outcome.
Freestone, 2012	Health Information Management Journal	Aus	All reportable	To examine use and quality of geographic location data in the NCIS.	Retrospective case series	6	National	All	1	NCIS data can be used at high levels of granularity. Data cleaning can be used to improve data quality.
Guiney, 2012	Australian Journal of Rural Health	Aus	Intentional self-harm	To determine whether farming suicides increased in Victoria during the prolonged drought.	Retrospective case series	7	VIC	Farmers	1	Due to the small number of relevant cases, there was no concluding evidence of a pattern of increasing farming suicides during the drought years.
Jones 2012[1]	International Journal of Injury Control and Safety Promotion	UK	Work-related deaths	To describe the circumstances of work-related fatalities in workers aged 55 years and older.	Retrospective case series	10	National	55 years and older	1	Most fatalities were male, working in agriculture, forestry and fishing, and were transport-related.
Judd 2012	Social Psychiatry and Psychiatric Epidemiology	Germany	Intentional self-harm	To examine the role of psychosocial factors, physical and mental health, gender in suicide.	Retrospective case series	5	National	All	1	Psychosocial factors were important, psychiatric morbidity was less than generally reported, and comparable proportions of males and females used violent means to suicide.

Author, Year	Journal	Country of Journal	Death Type	Aim	Study Design	Study Period	Jurisdiction	Population	NCIS as Data Source	Main Findings
Lippman 2012	Medical Journal of Australia	Aus	Recreational deaths	To examine the frequency and causes of snorkelling-related deaths.	Retrospective case series	7	National	All	1	Snorkelling-related deaths are rare. Risk factors include cardiac or respiratory disease, family history of sudden unexpected death, unfamiliarity with equipment and hyperventilation before breath-hold diving.
Lower 2012	NSW Public Health Bulletin	Aus	Injury-related deaths	To describe the pattern of non-intentional farm-related fatalities.	Retrospective case series	4	National	Farmers	1	The incidence of farm fatalities has declined. Most are transport-related, half are nonwork-related.
Machlin 2012	Crisis	Germany	Intentional self-harm	To determine the factors associated with an individual suicide featuring in the media.	Retrospective case series	1	National	All	Dual 1	Reported suicides tend to be those that may either heighten the risk of lethal imitative behaviors or serve to distort public perceptions about suicide.
Pilgrim 2012	Forensic Science International	Ireland	Poisoning deaths	To examine four fatal cases of MDMA-moclobemide induced serotonin toxicity.	Retrospective case series	7	VIC	All	1	Symptoms included hyperthermia, hyperkalemia, profuse sweating, twitching and shaking. Two cases involved concentrations associated with toxicity, and three some form of heart disease.
Pilgrim 2012	Current Drug Metabolism	United Arab Emirates	Poisoning deaths	To genotype the six main CYP450 enzymes in fatalities where target drugs susceptible to metabolic variation were detected.	Retrospective case series	4	VIC	All	1	Variation in genotype for rate of metabolisation varied for different enzymes.
Routley 2012	International Journal of Injury Control and Safety Promotion	UK	Work-related intentional self-harm	To examine work related suicides recorded on the Victorian Work Related Fatality Database (VWRFD).	Retrospective case series	8	VIC	All	2	Half of cases had an association with work stressors including business difficulties, work injury, unemployment/redundancy or conflict with supervisors/colleagues.
Sveticic 2012	BMJ Open	UK	Intentional self-harm	To compare the characteristics of persons who die by suicide	Retrospective case series	8	QSL	All	Dual 1	Suicides by missing persons show several distinct characteristics in

Author, Year	Journal	Country of Journal	Death Type	Aim	Study Design	Study Period	Jurisdiction	Population	NCIS as Data Source	Main Findings
				after being reported as missing compared with other suicides.						comparisons to other suicides in terms of method, location, physical health and communicating their intent.
Walter 2012[1]	Health and Place	US	All reportable deaths	To examine the relationship between remoteness of locations of deaths and coroners' decisions to hold inquests.	Retrospective case series	8	Multiple	All	1	The remoteness of a death's location strongly and positively predicted the chance that an inquest will be held.
Walter 2012[1]	Canadian Medical Association Journal	Canada	All reportable	To compare the characteristics of discretionary inquests and cases resolved through administrative investigations.	Retrospective case series	8	Multiple	All	1	Deaths that coroners choose to take to inquest differ systematically from those they do not in terms of age of the decedent and cause of death.
Cheung 2013	PLoS One	US	Intentional self-harm	To develop techniques in using scan statistics to detect clusters of suicide.	Retrospective case series	5	National	All	1	There were discrepancies between national and state/territory cluster detection by scan statistics due to the contrast of the underlying suicide rates across states/territories.
Kitching 2014[1]	International Journal of Injury Control and Safety Promotion	UK	Work-related deaths	To describe pedestrian fatalities in workplaces in relation to vehicles, mobile plant and their loads.	Retrospective case series	11	National	Pedestrian s	Dual 1	Most deaths occured in Agriculture and Construction, involving farmers, labourers and machinery operators.
Lower 2013	Australian and New Zealand Journal of Public Health	Aus	Quad bike-related deaths	To determine the economic costs of quad bike-related fatalities.	Retrospective case series	10	National	All	Dual 1	The estimated total economic cost associated with quad bike fatalities was $288.1 million, with an average cost for each fatality of $2.3 million.
Pilgrim 2013	Forensic Science, Medicine and Pathology	US	Poisoning deaths	To describe the circumstances and associated co-morbidities of quetiapine-related deaths.	Cohort study	9	VIC	All	1	Misuse of quetiapine was not a significant issue, and only occasionally led to fatalities when used in excess or concomitantly with interacting drugs
Pilgrim 2013	Forensic Science	Ireland	Poisoning	To determine the presence and	Cohort study	12	VIC	All	1	Cocaine was detected relatively

Author, Year	Journal	Country of Journal	Death Type	Aim	Study Design	Study Period	Jurisdiction	Population	NCIS as Data Source	Main Findings
	International		deaths	involvement of cocaine in sudden and unexpected death.						infrequently. However it appeared to be associated with a significant degree of cardiotoxicity, independent of cocaine concentration.
Pridmore 2013	Asian Journal of Psychiatry	Netherlands	Intentional self-harm	To apply the OPS framework to coroners' reports to compare suicides in Indigenous and non-Indigenous communities.	Retrospective case series	11	NT	Indigenous	1	The non-Indigenous group was over represented in the mental illness category and the Indigenous group was over represented in the social/environmental category.
Roxburgh, 2013	Drug Alcohol Review	UK	Poisoning deaths	To describe trends in fentanyl prescribing and mortality rates of fentanyl-related deaths.	Retrospective case series	13	National	All	Dual 1	A significant increase in fentanyl prescribing in Australia was observed as was an increase in fentanyl deaths.
Anthikkat 2013	Journal of Paediatrics and Child Health	Aus	Transport-related deaths	To investigate environmental and socio-demographic risk factors associated with low-speed vehicle run over fatalities in children aged 0–5 years.	Retrospective case series	11	National	Children	1	The incidence was low and declined over the study period. Most incidents occurred in non-traffic settings, with vehicles most likely to be reversing, and more commonly in lower socio-economic status areas.
Bambach 2013	Journal of Transportation Safety & Security	UK	Transport-related deaths	To examine the crash mechanics of fatal motorcycle–barrier crashes.	Retrospective case series	6	National	All	Dual 1	Certain motorcycle types, crash postures, pre-crash speeds, impact trajectory angles can be used to inform motorcycle-barrier crash test protocols.
Brighton 2013	Natural Hazards and Earth System Sciences	Germany	Drowning deaths	To describe the number and demographics of rip current related drowning deaths and rescues.	Retrospective case series	8	National	All	Dual 1	Rip currents were related to 44% of all beach-related drowning deaths and 57.4% of reported major rescues which is much lower than traditionally cited.
Ehsani 2013[1]	Safety Science	Netherlands	Work-related deaths	To describe fatal incidents involving young workers aged 15–24-years.	Retrospective case series	8	National	People aged 15-24 years	1	The Agriculture, Forestry and Fishing sector had the highest fatality rate for young workers,

Author, Year	Journal	Country of Journal	Death Type	Aim	Study Design	Study Period	Jurisdiction	Population	NCIS as Data Source	Main Findings
										and fatality rates were higher than the overall rate.
Goeman 2013	BMJ Open	UK	Asthma-related deaths	To characterise the current circumstances surrounding the asthma deaths and to identify potential interventions.	Retrospective case series	5	National	All	1	Risk factors included physical barriers, psychosocial issues, smoking, drug and alcohol dependence, allergies, respiratory tract infections, inadequate treatment and delay in seeking help.
Grzebieta 2013	International Journal of Crashworthiness	UK	Recreational deaths	To examine the pattern of hospitalised morbidity and death associated with go-kart-related-injuries.	Retrospective case series	8	National	All	1	In-depth analysis of go-kart fatal incident can be used to design crashworthy vehicle and barrier systems.
Grzebieta 2013	Journal of the Transportation	US	Transport-related deaths	To investigate the crash mechanics and injury profiles of 78 fatal motorcycle–barrier crashes.	Retrospective case series	6	National	All	1	Half the motorcyclists were in the upright posture, the mean precrash speed was 100.8 km/h, and thorax area had the highest incidence of injury.
Lingard 2013	Engineering, Construction and Architectural Management	UK	Work-related deaths	To investigate the causes of fatal incidents involving excavators in the Australian construction industry.	Retrospective case series	Unknown	National	All	1	Causes included the use of unsafe work methods and suitability of plant. Communication and design of work processes, were likely contributors.
Lingard 2013	Engineering, Construction and Architectural Management	UK	Work-related deaths	To investigate the circumstances surrounding fatal incidents involving plant in the Australian construction industry.	Retrospective case series	Unknown	National	All	1	Most incidents involved excavators, trucks and cranes, and people being run over or struck by a moving object. Site layout and unsafe actions were the most commonly identified immediate circumstances.
Lippmann	Diving and Hyperbaric Medicine	Aus	Recretional deaths	To investigate diving-related fatalities recorded on the DAN Asia-Pacific (DAN AP) database.	Retrospective case series	1	National	All	1	Twelve deaths occurred while snorkelling, eight while scuba diving. Apnoeic hypoxia, cardiac-related issues were implicated.
Porter 2013	Australian & New	Aus	Deaths in	To analyse Indigenous deaths	Retrospective	9	National	Indigenous	Dual 1	Key contributing factors included

Author, Year	Journal	Country of Journal	Death Type	Aim	Study Design	Study Period	Jurisdiction	Population	NCIS as Data Source	Main Findings
	Zealand Journal of Criminology		custody	in police custody since the 1991 Royal Commission.	case series					alcohol, criminal behaviour and mental health.
Arnautovska 2014	Social Psychiatry and Psychiatric Epidemiology	Germany	Intentional self-harm	To describe farmer suicide in Queensland.	Retrospective case series	10	QSL	Farmers	Dual 1	No significant associations were found between rates of farmer and non-farmer suicide, or between the proportion of farmers in a region and farmer suicide rates.
Bambach 2014	Traffic Injury Prevention	US	Transport-related deaths	To describe the nature and causes of head and spine injuries sustained by motorcyclists in head-leading collisions with fixed objects.	Retrospective case series	13	National	All	Dual 1	There was a predominance of basilar skull fractures, and upper cervical spine injuries, indicating a typical impact orientation of sliding in the prone position with head extension.
Cantwell 2014	Injury-International Journal of the Care of the Injured	UK	Pneumothorax	To identify temporal trends in the number of needle decompressions and untreated patients, and any unintended adverse consequences from the revised CPG.	Retrospective case series	4	VIC	All	Dual 1	There was a decrease in untreated tension pneumothoraces, but an increase in the number of patients that needed further treatment on arrival.
Dodds 2014	Health Information Management Journal	Aus	Intentional self-harm	To explore the process and problems of coding of the NCIS database 'intent' fields, and assess the use an option-based checklist.	Retrospective case series	1	Multiple	All	1	Coroners and coders do not follow a standard proforma. Coders rely mainly on police report at case notification and coroners' findings at case completion.
Gabbe 2014	Annals of Surgery	US	Transport-related deaths	To describe the burden of road transport–related serious injury after the introduction of a regionalized, inclusive trauma system.	Retrospective case series	11	VIC	All	Dual 1	Incidence of transport-related deaths decreased whereas the incidence of hospitalized major trauma increased. Years of life lost decreased by 43%, and years lived with disability increased by 32%.
Gibb 2014	Construction Management and	UK	Work-related activities	To apply a holistic framework to explain the way that causal	Comparative studies	11	National	All	Dual 1	The results suggest that the Construction Accident Causality

Author, Year	Journal	Country of Journal	Death Type	Aim	Study Design	Study Period	Jurisdiction	Population	NCIS as Data Source	Main Findings
	Economics			pathways to accidents occur.						framework is helpful for the analysis of the causes of accidents with outcomes of differing severity.
Law 2014	Social Psychiatry and Psychiatric Epidemiology	Germany	Intentional self-harm	To examine the socioeconomic and demographic characteristics of second-generation migrants who died by suicide.	Retrospective case series	8	Multiple	Migrants	Dual 1	Suicide in second-generation migrants tended to be represented by younger subjects, more often never married. Males aged 25-39 years tended to have a higher suicide risk than first-generation.
Lockley 2014	Suicide and Life-Threatening Behaviour	US	Intentional self-harm	To present the Masterplan project as a case study of a comprehensive response to suicide at a site known to be a hotspot for jumping suicides.	Retrospective case series	11	NSW	All	Dual 1	There were reductions in reported jumps and confirmed suicides, although the trends are not statistically significant, and a significant increase in police call-outs to intervene with suicidal people who have not yet reached the cliff's edge.
Milner 2014	International Journal of Epidemiology	UK	Intentional self-harm	To assess the association between labour force status and suicide with a particular focus on the unemployed, the economically inactive who theoretically are able to work and the employed.	Retrospective case series	10	National	All	1	Economically inactive/unemployed people had a greater suicide rate ratio than employed males people. There was an increase in suicide among both employed and economically inactive/unemployed people.
Milner 2014	Australian & New Zealand Journal of Psychiatry	Aus	Intentional self-harm	The present study focuses on the potential risks associated with the effects of involuntary job loss, as distinct from investigating the category of unemployment.	Retrospective case series	6	NSW	All	1	Involuntary job loss, low socio-economic status, mental disorder were associated with a greater odds ratio for suicide and attempted suicide.
Nott 2014	Australasian Journal on Ageing	Aus	Traumatic brain injury-related deaths	To examine the association between increasing age and mortality in TBI.	Retrospective case series	8	NSW	All	Dual 1	After controlling for known mortality risk factors, older patients were three times more

Author, Year	Journal	Country of Journal	Death Type	Aim	Study Design	Study Period	Jurisdiction	Population	NCIS as Data Source	Main Findings
										likely to die than younger patients. Crude mortality rates increased exponentially with advancing age.
Page 2014	BMC Psychiatry	UK	Intentional self-harm	To investigate the association between psychiatric disorders, socio-economic measures and suicide.	Retrospective case series	6	NSW	All	1	Strong associations were evident between mental disorders and suicide, especially for anxiety disorders. Associations for socio-economic status were smaller than for mental disorders.
Pilgrim 2014	Drug and Alcohol Dependence	Switzerla nd	Assault	To ascertain the prevalence of deaths in Australia involving "king hits" and determine the involvement of drugs and other sociological factors in these violent fatalities.	Retrospective case series	13	National	All	1	90 cases were identified with a median age of 33 years, 53 cases involved the use of alcohol or other drugs.
Pilgrim 2014	Forensic Science International	Ireland	Poisoning deaths	To determine the prevalence, blood concentrations and contribution of duloxetine.	Retrospective case series	4	VIC	All	1	Death involving duloxetine alone was uncommon; however with certain comorbidities and co-administered drugs, the risk of a fatal event is increased, particularly in the setting of other pro-serotonergic agents.
Clapperton 2013	Medical Journal of Australia	Aus	Quad bike-related deaths	To examine fatal and hospital-treated injury associated with quad bike admissions and ED presentations in Victoria.	Retrospective case series	10	VIC	All	Dual 1	The peak age group for fatalities and admissions was 15-29 years. Males were strongly overrepresented. Intracranial injury, fractures and traumatic asphyxiation were the most common injuries among fatal cases.

[1] **Publications involving authors of the present study.**

Legend

Country of journal: Aus = Australia; UK = United Kingdom; US = United States.

Jurisdiction: VIC = Victoria; WA = Western Australia; NT = Northern Territory; QSL = Queensland; NSW = New South Wales.

NCIS as Data Source: 1 = primary; 2 = secondary.
